# Synthesis, Electrical Conductivity, and Wave-Absorption Performances of Bamboo-Based Composites Co-Doped with Graphene Oxide and Polyaniline

**DOI:** 10.3390/polym17010078

**Published:** 2024-12-31

**Authors:** Jin Wang, Wangjun Wu, Wenfu Zhang, Ying Zhao, Hongyan Wang, Shaofei Yuan, Jian Zhang

**Affiliations:** 1 Key Laboratory of Bamboo Research of Zhejiang Province, Zhejiang Academy of Forestry, Hangzhou 310023, China; wangjin@zjforestry.ac.cn (J.W.); wwj18351869585@163.com (W.W.); zhangwenfu542697@163.com (W.Z.); zhaoyingabm@163.com (Y.Z.); 15990054143@163.com (H.W.); 2 College of Chemistry and Materials Engineering, Zhejiang Agricultural and Forestry University, Hangzhou 311300, China

**Keywords:** carbonized bamboo, polyaniline, graphene oxide, microwave absorption, conductivity, composite materials

## Abstract

Bamboo was carbonized and further modified via co-doping with graphene oxide (GO) and polyaniline (PANI) to prepare microwave absorption composites (GO/PANI/CB) by in situ polymerization of 1R-(-)-Camphorsulfonic acid (L-CSA). The conductivity of GO/PANI/CB reached 2.17 ± 0.05 S/cm under the optimized process conditions. The oxygen-containing group of GO reacts with PANI to form hydrogen bonds and thus polymerize. The GO and PANI particles covered the carbonized bamboo (CB) surface in a disordered aggregation form. Based on the measuring method of the vector network analyzer (VNA), the microwave-absorption performance of GO/PANI/CB was investigated. With 30% addition of GO/PANI/CB, the minimum reflection loss (RL_min_) at 7.12 GHz with a thickness of 3.5 mm of samples reached −49.83 dB. The effective absorption bandwidth (<−10 dB) is as high as 4.72 GHz with a frequency range of 11.68–16.40 GHz and a thickness of 2 mm. Compared with many PANI based electromagnetic wave absorbing materials reported in recent years, GO/PANI/CB provides improved microwave-absorption performance while maintaining high absorption bandwidth. GO/PANI/CB exhibited the advantages of simple preparation, low cost, renewability, light texture, thinness, wide absorption bandwidth, and strong absorption ability, and can be used for new microwave absorption materials.

## 1. Introduction

Nowadays, electronic devices and communication technologies that use electromagnetic waves as carriers are developing rapidly. However, the resulting electromagnetic pollution problem is becoming increasingly prominent, not only affecting the high precision and reliability of electronic devices, but also seriously damaging human health. Controlling or mitigating the hidden dangers of electromagnetic pollution has become a pressing issue faced by countries around the world [1,2,3,4,5]. At present, there are mainly two effective methods to deal with electromagnetic pollution, namely electromagnetic shielding and microwave absorption. Electromagnetic shielding cannot fundamentally eliminate electromagnetic waves. Microwave absorbing materials fundamentally mitigate electromagnetic pollution by converting electromagnetic waves into other forms of energy, such as heat [6,7,8]. Therefore, more and more researchers are committed to developing microwave absorption materials to reduce the impact of electromagnetic wave pollution. The thinness, light weight, strong absorption band, and stability of microwave absorption materials are research hotspots.

The existing microwave absorption materials include magnetic materials such as ferrum (Fe) [9,10,11], nickel (Ni) [12,13], and cobalt (Co) [14,15], as well as dielectric materials such as barium titanate (BaTiO_3_) [16,17], carbon materials [18,19], silicon carbide (SiC) [20], graphene [21], and conductive polymers [22]. However, the high cost, high density, poor biodegradability, and processing difficulties of the above materials greatly limit their application. In the context of the global low-carbon economy [23], there is an urgent need to research new microwave absorbing materials that are lightweight, high-yielding, biodegradable, and easy to process. Wood or bamboo timber is expected to be an ideal material in the presence of its renewable, recyclable, and degradable characteristics. With the sharp decline in global forest area, bamboo is also gradually becoming an effective alternative to wood and plastic. CB, as a preliminary product of bamboo processing, has the advantages of renewable, high yield, and low cost, as well as unique advantages. CB has certain electrical properties and a large specific surface area, which allows it to interact with electromagnetic waves and consume them. The porous structure of CB gives it certain microwave absorption properties and serves as an efficient carrier for various absorbents.

In recent years, many high-quality absorbents have been developed and reported, including transition metal carbides and nitrides (MXenes) [24], graphene and its derivatives [25], carbon nanotubes (CNTs) [26], carbon nanocoils (CNCs) [27], silicon carbide (SiC) [28], and conductive polymers [29]. Among them, PANI is one of the most concerning dielectric loss absorbents due to its high chemical stability, low density, simple preparation, high bio-compatibility, and controllable conductivity after doping [30,31]. Although doped PANI has high conductivity, severe impedance mismatch often limits its absorption performance. Therefore, L-CSA was chosen as the doping acid, which not only promoted the dispersion of PANI as a surfactant but also enabled the doped PANI to have higher electrical conductivity and better impedance matching. GO exhibits outstanding hydrophilicity, wave-transmitting property, a large specific surface area, and stable physical and chemical properties. Therefore, many researchers have started to study GO-based electromagnetic wave absorbing materials and revealed their electromagnetic wave absorbing principles, including conductivity loss and polarization loss [32,33]. GO is the perfect composite template material to effectively alleviate the impedance mismatch of PANI [34]. There are many studies on GO/PANI composite wave absorbents, mainly focusing on the composition design of magnetic components [35]. The effect of polyaniline grafting on magnetite functionalized with aminopropy ltrimethoxysilane was studied for the PANI/ferrite composites, and the correlation between the maximum grafting probability and the maximum magnetization was evidenced [36]. Zhao [37] et al. prepared PANI/GO/Fe_3_O_4_ ternary microwave absorption composites with RL_min_ of −53.5 dB. Liu [38] et al. designed magnetic graphene@PANI@porous TiO_2_ ternary hetero-structure wave microwave absorption composites, achieving the RL_min_ of −45.4 dB. However, the complex operational steps and high density have not fully utilized the advantages of simple preparation and lightweight texture of GO/PANI.

In this paper, the inter-facial polymerization between the composite absorbent particles of CB, GO and L-CSA-doped PANI was investigated. Combining their advantages, a bamboo based composite microwave absorbing material has been prepared, which is simple to prepare, low-cost, renewable, lightweight, thin in thickness, has a wide absorption bandwidth, and strong absorption ability. Meanwhile, the structure, composition, and electromagnetic properties of the new composite materials were investigated. The formation mechanism was revealed to provide theoretical support for the development and application of microwave absorption composites.

## 2. Materials and Methods

### 2.1. Materials and Instruments

The 5-year-old moso bamboo was harvested from Suichang County, Zhejiang Province, and processed into slices with a moisture content of approximately 10%. GO was purchased by Tanfeng Technology Co., Ltd. (Suzhou, China). Aniline and ammonium persulfate (APS) were purchased by Colon Chemical Co., Ltd. (Chengdu, China). L-CSA was purchased by Shanghai Maclin Biochemical Technology Co., Ltd. (Shanghai, China). The electronic balance (BSA124S, Max120 g, d = 0.1 mg) was produced by Certoris Scientific Instrument Co., Ltd. (Beijing, China) The magnetic heating agitator (HJ-6) was provided by the Jintan Chengdong Xinrui Instrument Co., Ltd. (Changzhou, China). The ultrasonic cleaning instrument was obtained from the Jiemeng Cleaning Equipment Co., Ltd.(Shenzhen, China) The drying oven was produced by Boxun Industrial Co., Ltd. (Shanghai, China). The box resistance furnace (SX2-5-12) was supplied by the Experimental Electric Furnace Co., Ltd. (Shanghai, China).

### 2.2. Preparation of CB

The bamboo pieces were cleaned with ultrasonic waves and then dried continuously at 103 °C for 12 h. The bamboo pieces were ultrasonically cleaned and then dried at 103 °C for 12 h. The above bamboo pieces were placed in a porcelain boat in a resistance furnace for high-temperature carbonization. The process followed a programmed temperature increase, rising from ordinary temperatures to 800 °C at a rate of 10 °C/min and maintaining at 800 °C for 5 h. The furnace was cooled to room temperature and protected with nitrogen throughout the entire carbonization process. CB was obtained by grinding bamboo charcoal in a mortar and pestle. The CB was washed with deionized water, then filtered and dried.

### 2.3. Synthesis of GO/PANI/CB

**Figure 1:**
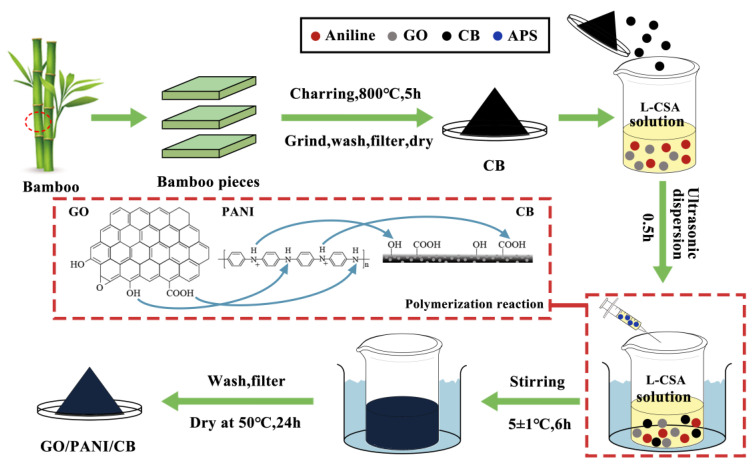
Schematic illustration of the synthesis and polymerization process of GO/PANI/CB.

A total of 90 mg of GO was ultrasonically dispersed in 30 mL of deionized water to form a 3 g/L mass concentration of GO dispersion.3 mL of aniline was mixed with 30 mLL-CSA solution (1 mol/L), and was stirred with an electromagnetic stirrer for 0.5 h. 3 g of CB was added into the mixture along with the GO dispersion. After following ultrasonication for 0.5 h, the mixture was immersed in an ice bath maintained at 5 ± 1 °C. 4.56 g of APS (1 mol/L) was dissolved in 20 mL of L-CSA solution by adding dropwise to the mixture over 6 h with electromagnetic stirring in an ice bath. When finished, the samples were washed and filtered using deionized water and anhydrous ethanol. The as-prepared sample was L-CSA-modified GO/PANI/CB. The preparation process is illustrated in Figure 1. The L-CSA doped polyaniline sample without GO was used as the control, designated as PANI/CB.

Fix other experimental conditions and change the amount of GO added separately. Prepare samples with GO contents of 30 mg, 60 mg, 120 mg, and 150 mg, and label them with GO-30, GO-60, GO-120, and GO-150, respectively. Fix other experimental conditions and change the molar concentration of L-CSA changed. Prepare samples with L-CSA concentrations of 0.25 mol/L, 0.5 mol/L, 0.75 mol/L, 1.25 mol/L, and 1.5 mol/L, and label them with L-CSA-0.25, L-CSA-0.5, L-CSA-0.75, L-CSA-1.25, and L-CSA-1.5, respectively. Fix other experimental conditions and change the amount of CB added also changed. Prepare samples with CB contents of 1 g, 2 g, 4 g, 5 g, and 6 g, and label them with CB-1, CB-2, CB-4, CB-5, and CB-6, respectively. Fix other experimental conditions and change APS concentration. Prepare samples with APS concentrations of 0.25 mol/L, 0.5 mol/L, 0.75 mol/L, 1.25 mol/L, and 1.5 mol/L, and label them with APS-0.25, APS-0.5, APS-0.75, APS-1.25, and APS-1.5, respectively.

### 2.4. Performance Testing

Use a four probe resistance tester (M-3, Suzhou Electronics Co., Ltd., Suzhou, China) to test the conductivity (σ, S/cm). Three replicates were performed in each group.

Synchronous thermal analysis (TG-DSC, STA409PC, NETZSCH Co., Ltd., Selb, Germany) was used to determine changes in sample mass and relevant information on heat absorption and release. Heat 15 mg of sample powder at a rate of 10 °C/min in a nitrogen protected atmosphere, ranging from 30 °C to 900 °C, to study the thermal stability of CB, PANI/CB, and GO/PANI/CB. Three replicates were performed in each group.

The CB, PANI/CB, and GO/PANI/CB composite powders were uniformly mixed with paraffin wax in a mass ratio of 3:7, with the filler loading of 30%. Preparation of ring standard samples by hot pressing. The electromagnetic parameters and S-parameters were measured by a vector network analyzer (PNA-N5245A, Agilent Technologies Ltd., Santa Clara, CA, USA) in the test frequency band of 2–18 GHz.

### 2.5. Characterization

The surface morphology and surface chemical compositions of CB, PANI/CB, and GO/PANI/CB samples were characterized by SEM (Quanta 200, Fidacon Ltd., Columbia, MD, USA) and EDS (attached to the SEM). By XRD (D/MAX 2200, Rigaku, Japan), the crystalline structure of CB, PANI/CB and GO/PANI/CB samples was identified using Cu Kα radiation (λ = 1.5418Ā) under the conditions of 40 kV and 40 mA. The functional groups in bamboo based composite material samples were analyzed using Fourier transform infrared (FTIR) spectroscopy (Spectrum One, Perkin Elmer Ltd., Hopkinton, MA, USA). Surface elemental composition analysis was performed using an Al Ka monochromatic X-ray source based on X-ray photoelectron spectroscopy (XPS, K-Alpha 1063, Thermo Fisher Scientific Ltd., Waltham, MA, USA), where all binding energies were calibrated for the C1s peak (284.8 eV). The CB, PANI/CB, and GO/PANI/CB samples were characterized by FTIR, XRD, XPS, SEM, and EDS.

## 3. Results and Discussion

### 3.1. Conductivity Analysis

**Figure 2:**
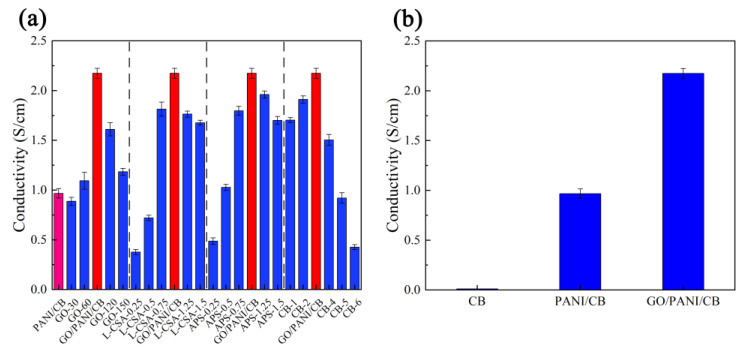
The conductivity of all samples (**a**), the conductivity of CB, PANI/CB, and GO/PANI/CB (**b**).

Based on the change in conductivity from PANI/CB to GO-150 samples, we can see the effect of GO on the samples (Figure 2 and Table 1). Compared with PANI/CB, the conductivity of the GO-30 sampled was decreased to 0.89 (±0.04) S/cm. When a small amount of GO was added, most oxygen-containing groups as an insulator in GO destroyed and replaced the conjugate structure of PANI [39]. At this time, GO was equivalent to a resistor to the conductive layer of PANI coated on the CB surface, which reduced the conductivity of the prepared samples. With the continuous increase in GO content, GO formed a conductive network in the PANI coated on the surface of CB, resulting in a significant increase in its conductivity. When the amount of GO was 90 mg, the conductivity of the prepared GO/PANI/CB samples reached a high level.

Based on the conductivity change from L-CSA-0.25 to L-CSA-1.5 samples, we can see the effect of L-CSA on the samples (Figure 2 and Table 1). With increasing L-CSA concentration, the conductivity increased first and then decreased. The polymerization of GO and An was well performed on the CB surface only under the condition of pH 2~3. An appropriate concentration of L-CSA in a certain range reduced the pH value of the reaction system, promoting the effective polymerization of GO and An on the CB surface. When the concentration of L-CSA reached 1.0 mol/L, the polymerization of GO and An on the CB surface and the conductivity were improved.

Based on the conductivity change from APS-0.25 to APS-1.5 samples, we can see the effect of APS on the samples (Figure 2 and Table 1). With increasing APS molar concentration, the conductivity increased first and then decreased. When the concentration of APS was too low, the in situ polymerization reaction between GO and An on the surface of CB was not sufficient. The resulting GO/PANI cannot completely coat CB or the coating layer was too thin, which reduced its conductivity. When the concentration of the oxidant APS was too high, the in situ polymerization of GO and An on the surface of CB had too many active centers accompanied by intense reactions, causing local oxidation of the aniline to generate smaller molecules [40]. Therefore, the uneven or more localized coating of the polymerized GO/PANI was formed on the CB surface, resulting in shedding and reduced conductivity. When the molar concentration of APS was 1 mol/L, GO and PANI underwent effective polymerization on the surface of CB, improving conductivity.

Based on the conductivity change from CB-1 to CB-6 samples, we can see the effect of CB on the samples (Figure 2 and Table 1). With increasing CB content, the conductivity increased first and then decreased. When the CB content was low, the surface of CB was covered with more GO/PANI. The GO/PANI layer tended to peel off, resulting in an uneven conductive layer, and further affecting the conductivity of the prepared samples. As the CB content increased, an appropriate amount of CB was uniformly and densely encapsulated by GO/PANI, improving the electrical conductivity of the prepared samples. With the continuous increase in CB content, the GO/PANI produced by the polymerization reaction cannot completely cover the excess CB, which affected the conductivity of the prepared samples. When the CB content was 3 g, the conductivity of GO/PANI/CB reached a high level.

Figure 2b shows the conductivity of CB, PANI/CB, and GO/PANI/CB. The conductivity of CB was less than 0.01 S/cm (the minimum range of the four-probe resistance tester used in this experiment is 0.01 S/cm. The conductivity of the PANI/CB and GO/PANI/CB samples was 0.97 (±0.05) S/cm and 2.17 (±0.05) S/cm, which was higher than that of CB, indicating that the addition of PANI and GO improved the conductivity of CB.

In summary, the optimal preparation process of GO/PANI/CB composite is shown as 90 mg of GO addition, 1.0 mol/L of L-CSA concentration, 1.0 mol/L of APS concentration, and 3 g of CB addition. At this time, the conductivity of GO/PANI/CB reaches the optimum, which is 2.17 (±0.05) S/cm. The optimal conductivity of GO/PANI/CB does not reach the range of a conductor, which is a semiconductor material. Under this condition, the insulation performance of GO/PANI/CB is better, which lead to reducing the formation of surface reflection waves on the material and making it positively correlated with electromagnetic wave absorption performance [41]. Therefore, the preparation process of GO/PANI/CB is optimized to enhance the conductivity of the material and at the same time enhance the electromagnetic wave absorption performance of the material.

### 3.2. FTIR Analysis

**Figure 3:**
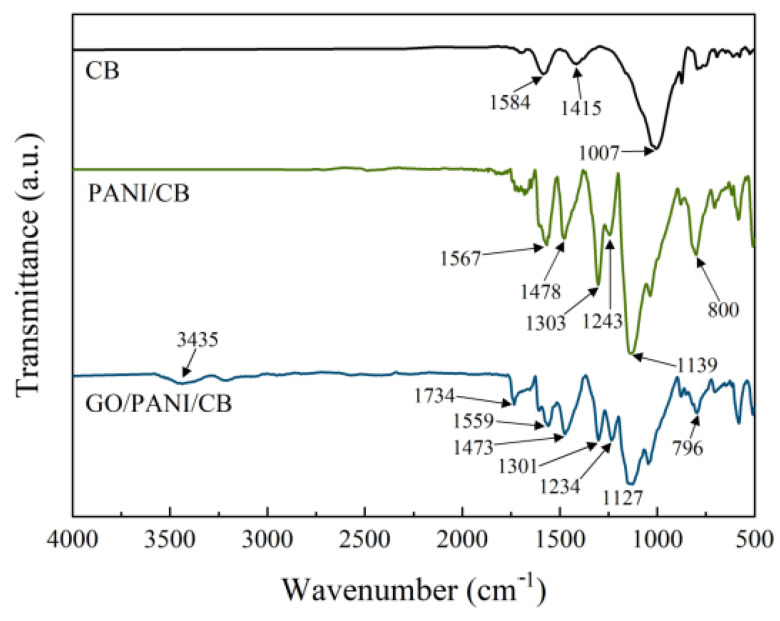
FTIR spectra of GO/PANI/CB, PANI/CB, and CB.

The FTIR spectra of GO/PANI/CB, PANI/CB, and CB are shown in Figure 3. In the FTIR spectrum of CB, the peak at 1584 cm^−1^ is attributed to the C-C stretching vibration peak in the aromatic ring skeleton, the peak at 1415 cm^−1^ is attributed to the O-H bending vibration peak, and the peak at 1007 cm^−1^ is related to the C-O stretching vibration peak in the alcohol hydroxyl group. There are few functional groups on the surface of CB, only C-C bonds and hydroxyl functional groups.

In the FTIR spectrum of PANI/CB, the peak at 1567 cm^−1^ corresponds to the stretching vibration peak of the C=C bond in the quinone structure N=Q=N, the peak at 1478 cm^−1^ is attributed to the stretching vibration peak of the C=C bond in the benzene structure N=B=N the peak at 1303 cm^−1^ comes from the bending vibration peak of the C-N bond in aromatic amines, the main peak at 1243 cm^−1^ is attributed to the stretching vibration peak unique to the PANI L-CSA doped state, the peak at 1139 cm^−1^ is attributed to the C-H bond, and the peak at 800 cm^−1^ is caused by the out of plane stretching vibration peak of the C-H bond. The FTIR spectrum of PANI/CB is consistent with the molecular structure of polyaniline doped state in previous studies [42], indicating the presence of PANI and its polymerization on the CB surface in PANI/CB.

The spectrum of GO/PANI/CB shows that the stretching vibration peak of hydroxyl groups in oxidized graphene is located at 3435 cm^−1^, and the stretching vibration peak of C=O bonds in oxidized graphene is located at 1734 cm^−1^. The 1559 cm^−1^ is the stretching vibration peak of the C=C bond in the quinone structure N=Q=N [43]. In addition, the stretching vibration peak of the C=C bond in the benzene structure N=B=N is located at 1473 cm^−1^, and the bending vibration peak of the C-N bond in aromatic amines is located at 1301 cm^−1^. The stretching vibration peak unique to the L-CSA doped state of PANI is concentrated at 1234 cm^−1^, the in-plane stretching vibration peak of the C-H bond is located at 1127 cm^−1^, and the out of plane stretching vibration peak of the C-H bond is around 796 cm^−1^. These characteristic peaks are basically consistent with PANI/CB, but new characteristic peaks also appear at 3435 cm^-1^ and 1734 cm^−1^. The results indicate that the addition of GO affects the molecular structure of PANI. The GO and PANI copolymerize on the surface of CB to form a coating on CB.

### 3.3. XRD Analysis

**Figure 4:**
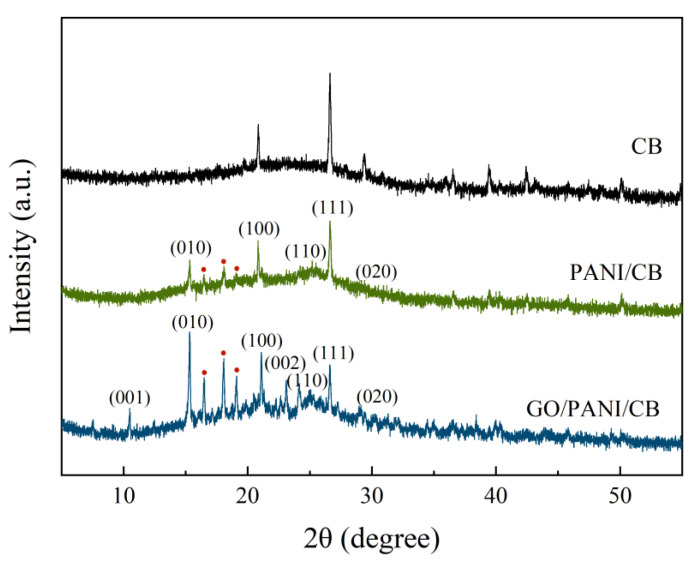
XRD patterns of GO/PANI/CB, PANI/CB, and CB.

The XRD patterns of CB, PANI/CB, and GO/PANI/CB are shown in Figure 4. The XRD curve of CB shows multiple peaks. This is because CB is carbonized from bamboo, which absorbs a large amount of minerals from the soil during its growth process. Even after carbonizing, these minerals are still retained. Therefore, CB not only contains organic compounds such as C, H, O, and N, but also minerals such as Mg, K, and Na. Therefore, the XRD curve of CB has multiple peaks.

The diffraction peaks of PANI/CB appeared at approximately 15.3°, 20.8°, 25.2°, 26.6°, and 30.5°, corresponding to the (010), (100), (110), (111), and (020) crystal planes of the doped PANI, respectively, which were consistent with a previous study reported in the literature on PANI [44,45]. The diffraction peaks of GO/PANI/CB were located at approximately 15.3°, 21.1°, 25.1°, 26.6°, and 30.3°, deriving from the (010), (100), (110), (111), and (020) crystal planes of the doped PANI, respectively, which basically coincided with the polyaniline diffraction peak of PANI/CB. This indicated the presence of PANI in both GO/PANI/CB and PANI/CB. The diffraction peaks on the crystalline surfaces of (110) and (020) were not obvious, which was due to the dispersion of the PANI system caused by L-CSA as a dopant acid and a surfactant.

By comparing the three spectra of the CB, PANI/CB, and GO/PANI/CB samples, it can be found that the three diffraction peaks (red dot Figure 4) representing PANI appear at 2θ = 15°–20°. The diffraction peaks of GO/PANI/CB are sharper than those of PANI/CB. The appearance of characteristic peaks here indicates that PANI in GO/PANI/CB composite materials is more crystalline, with molecular chains arranged along specific directions. During the polymerization process, GO can induce PANI crystallization and orderly stacking in specific directions, thus increasing the degree of crystallinity. The increase in crystallinity means an increase in conductivity, which can be used in composite materials to provide improved conductivity, consistent with conductivity.

There are two new diffraction peaks of GO/PANI/CB near 10.5° and 24.2° for the (001) and (002) crystal planes of GO [46], which proved the presence of GO in GO/PANI/CB. The diffraction peaks on the (010) and (100) crystal planes in GO/PANI/CB were significantly higher than those in PANI/CB, which demonstrated that the interaction between GO and PANI reduced the crystallinity and particle size of PANI [47].

### 3.4. XPS Analysis

**Figure 5:**
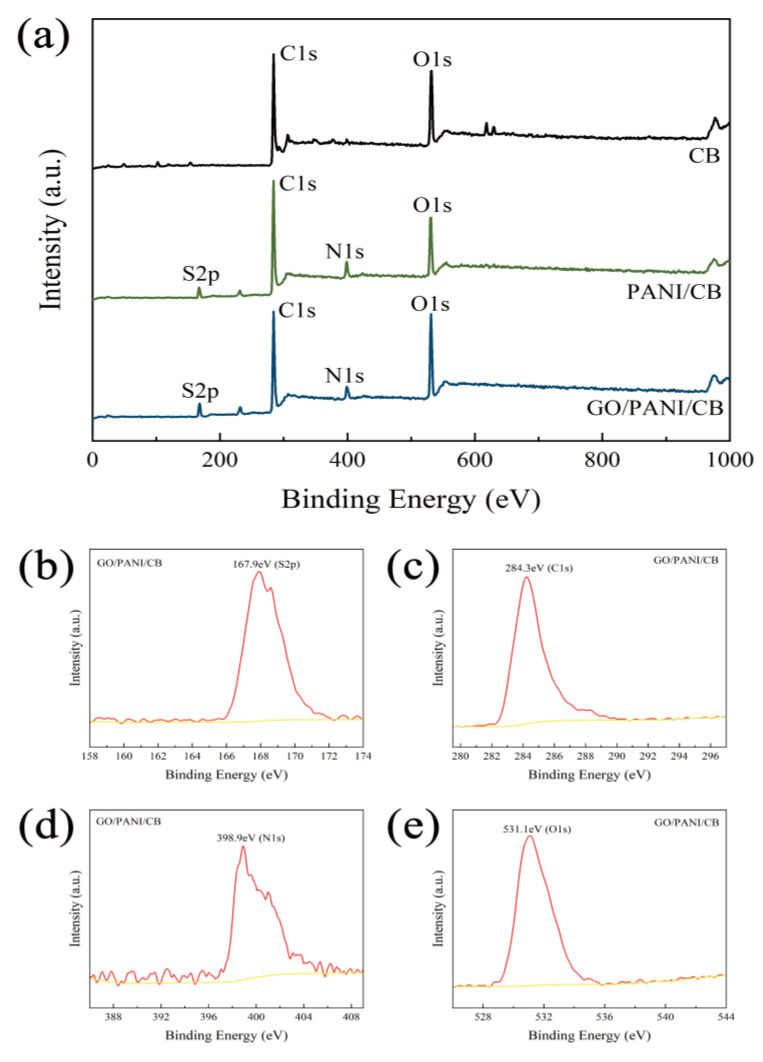
XPS spectra of GO/PANI/CB, PANI/CB, and CB (**a**); S_2p_ (**b**), C_1s_ (**c**), N_1s_ (**d**), and O_1s_ (**e**) regions of GO/PANI/CB.

The XPS spectra of CB, PANI/CB, and GO/PANI/CB samples are shown in Figure 5a. There were the characteristic peaks of C_1s_, N_1s_, O_1s_, and S_2p_ shown in the spectra of GO/PANI/CB and PANI/CB, indicating the presence of C, N, O, and S in GO/PANI/CB and PANI/CB. CB possessed only the C_1s_ and O_1s_ characteristic peaks. The S_2p_ characteristic peak was derived from the sulfur element of L-CSA, and the N1s characteristic peak was caused by the nitrogen element of aniline.

The intensity of the O_1s_ characteristic peak in GO/PANI/CB is higher than that in PANI/CB, due to the interaction between the oxygen-containing groups in GO and PANI. The results are consistent with those of FTIR and XRD. The binding energies of the S_2p_, C_1s_, N_1s_, and O_1s_ characteristic peaks of GO/PANI/CB shown in Figure 5b–e were approximately 167.9 eV, 284.3 eV, 398.9 eV, and 531.1 eV, respectively, which was in general agreement with the results reported in the literature from previous studies of PANI [44], proving the presence of L-CSA-doped PANI in GO/PANI/CB.

### 3.5. SEM Analysis

**Figure 6:**
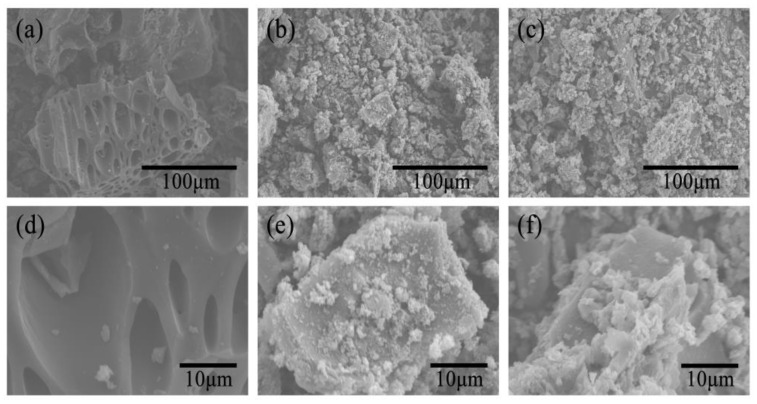
SEM images of CB (**a**,**d**), PANI/CB (**b**,**e**), and GO/PANI/CB (**c**,**f**).

As shown in Figure 6a,d, the original CB surface was relatively clean, exhibiting a large number of pore structures of different sizes, which facilitated the polymerization of PANI and GO/PANI on the CB surface and in the pores. As shown in Figure 6b,e, the PANI particles of different sizes are randomly dispersed on the surface of CB, making the CB surface rough, indicating successful polymerization of PANI on CB. With the addition of GO, GO interacts with PANI and polymerizes on the surface and pores of CB, resulting in GO/PANI/CB, as shown in Figure 6c,f. The GO/PANI particles covered the surface of CB in the form of disordered agglomerates. The GO/PANI particles were larger in size and more tightly polymerized on CB than PANI, which was the template role of GO for the growth of PANI in two dimensions.

### 3.6. EDS Analysis

**Figure 7:**
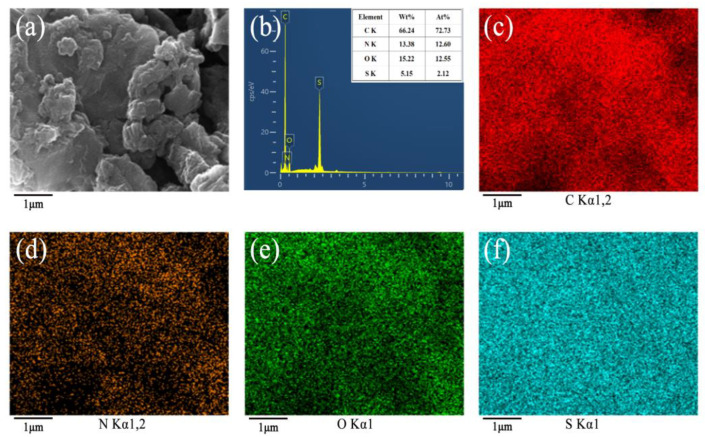
SEM images of GO/PANI/CB (**a**) and EDS spectra of GO/PANI/CB (**b**). The inset tables present the element contents. C (**c**), N (**d**), O (**e**), and S (**f**) element mapping images of GO/PANI/CB.

Figure 7a shows a high-magnification electron microscope scan of the EDS energy spectrum of GO/PANI/CB. The GO/PANI particles covered the surface of CB in the form of disordered clusters. Figure 7b shows the chemical elements and elemental content of GO/PANI/CB. There were N and S in GO/PANI/CB, which indicated the generation of PANI doped with L-CSA, as analyzed by the XRD and XPS spectra. The C, N, O, and S elemental mapping images of GO/PANI/CB are shown in Figure 7c–f. Element C had the largest area distribution, followed by elements N and O, and element S possessed the smallest area distribution, which also coincided with the elemental content of Figure 7b. The C, N, O, and S elements were well dispersed, which proved that GO/PANI was homogeneously agglomerated on the surface of the CB.

### 3.7. TG-DSC Analysis

**Figure 8:**
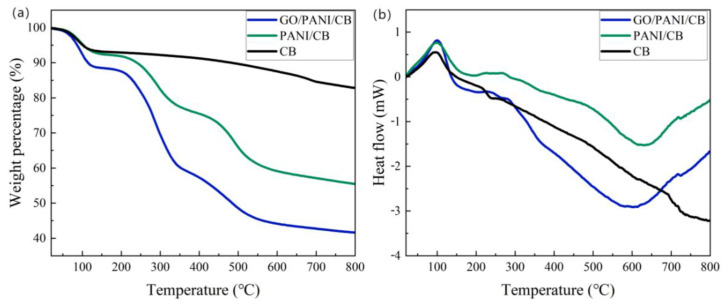
TG curves (**a**) and DSC curves (**b**) of GO/PANI/CB, PANI/CB, and CB.

As shown in Figure 8, all samples showed mass loss between about 60 and 120 °C, which was caused by water evaporation. The weight loss of GO/PANI/CB is about 10%, slightly higher than other samples, attributed to the hydrophilicity of GO and the slight hygroscopicity of residual L-CSA. The heating stage from 120 °C to 800 °C was the exothermic stage, and the weight loss of the CB sample was not significant during this stage, which was the removal of most cellulose, hemicellulose, and lignin during the previous carbonization process. At approximately 200 °C to 350 °C, the exothermic capacities of PANI/CB and GO/PANI/CB gradually increased, with a large and continuous loss of mass, which was attributed to the residual L-CSA starting to decompose by heat. In contrast, GO/PANI/CB had a higher mass loss than PANI/CB, which was caused by the decomposition of GO in GO/PANI/CB. At approximately 350 °C to 550 °C, the exothermic capacities of GO/PANI/CB and PANI/CB gradually increased, the mass loss continued, and the rates of loss were close, which was due to the decomposition of PANI at this stage. Between 550 °C and 800 °C, the exothermic capacities of GO/PANI/CB and PANI/CB decreased, and the mass loss was smaller, which indicated that the materials polymerized on CB were essentially completely decomposed, leaving behind CB.

The sample residues of GO/PANI/CB, PANI/CB, and CB at 800 °C were 40.9%, 54.5%, and 81.8%, respectively, which demonstrated that the amount of material polymerized on CB in GO/PANI/CB was higher than that in PANI/CB with the addition of GO.

### 3.8. Electromagnetic Properties Analysis

**Figure 9:**
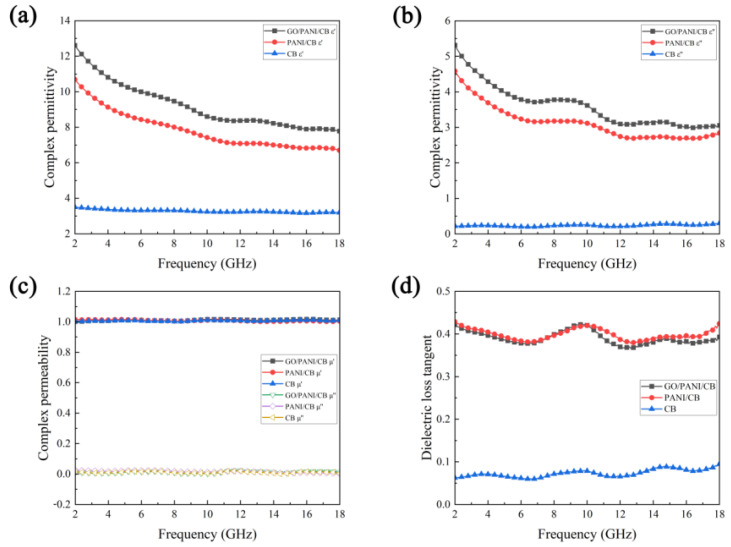
The frequency dependence of the real part (**a**) and imaginary part (**b**) of the complex permittivity and complex permeability (**c**) of GO/PANI/CB, PANI/CB, and CB, as well as the corresponding dielectric loss tangent (**d**).

Figure 9a,b show the real part (ε′) and imaginary part (ε″) of the dielectric constant of three samples in the range of 2–18 GHz. The real and imaginary parts of the dielectric constants of GO/PANI/CB and PANI/CB decrease with increasing frequency, exhibiting significant dielectric dispersion phenomena. The real and imaginary parts of the dielectric constant of GO/PANI/CB are higher than those of PANI/CB, which is consistent with the variation pattern of the conductivity measurement of powder compacts. The conductivity of GO/PANI/CB prepared under optimal process conditions is higher than that of PANI/CB, indicating that GO/PANI/CB has higher dielectric storage capacity and dielectric loss capacity than PANI/CB. The appropriate proportion of addition helps to form a better PANI conductive network on the CB surface. The large molecule camphor sulfonate (CSA -) in L-CSA doped simultaneously further enhances the carrier transport between GO and PANI coated on the CB surface through interactions such as electrostatic attraction or hydrogen bonding [48]. The real part of the dielectric constant (ε′) of CB is about 4, and the imaginary part (ε″) is slightly greater than 0, indicating that CB itself has a certain dielectric storage capacity and dielectric loss capacity.

In Figure 9c, the real part of the complex permeability of GO/PANI/CB, PANI/CB, and CB fluctuates about 1.0 with frequency, and the imaginary part of the permeability fluctuates about 0. This fluctuation is close to the value of paraffin matrix, indicating that the magnetic loss contribution of these three samples is very small. All three samples of GO/PANI/CB, PANI/CB, and CB are non-magnetic loss materials. Figure 9d shows the dielectric loss tangent lines (tanδE = ε″/ε′) of GO/PANI/CB, PANI/CB, and CB. The tanδE = ε″/ε′ represents the ratio of electromagnetic energy loss to storage, which is commonly used to determine the dielectric loss performance of absorbing materials. Generally, materials with better dielectric loss performance have better absorption performance. The dielectric loss tangent of CB remains around 0.05, almost close to 0. The dielectric loss tangent values of GO/PANI/CB and PANI/CB are significantly higher than CB, remaining around 0.4. This is consistent with the results of the conductivity test, indicating that the dielectric losses of GO/PANI/CB and PANI/CB are composed of conductivity losses and polarization losses. GO/PANI/CB and PANI/CB are both dielectric loss composite materials.

Relationship between dielectric loss and Debye polarization relaxation [49]:

(1) $${\left(\varepsilon^{\prime }-\frac{\varepsilon_{s}+\varepsilon_{\infty }}{2}\right)}^{2}+{\left(\varepsilon^{\prime \prime }\right)}^{2}={\left(\frac{\varepsilon_{s}-\varepsilon_{\infty }}{2}\right)}^{2},$$

**Figure 10:**
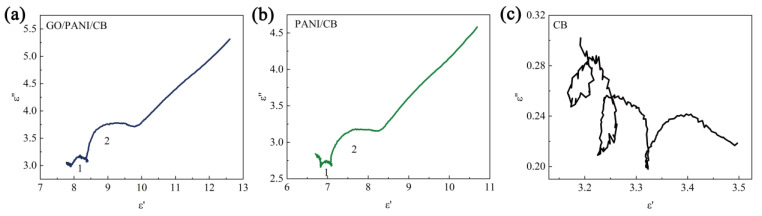
Cole-Cole semicircles of GO/PANI/CB (**a**), PANI/CB (**b**), and CB (**c**).

Among them, ε_∞_ and ε_s_, respectively, represent the dielectric constant and static dielectric constant at infinite frequency. The relationship diagram between ε_∞_ and ε_s_ should be a single semicircle defined as a Cole–Cole semicircle. Each semicircle corresponds to one Debye relaxation process [50]. As shown in Figure 10a,b, two semicircles appear in the curves of GO/PANI/CB and PANI/CB, indicating that the double Debye dipole relaxation process occurs in both GO/PANI/CB and PANI/CB [51,52]. It is shown that both samples possess excellent dielectric loss capability. As shown in Figure 10c, the Cole–Cole curve of CB displays semicircles with multiple small numerical spans, with an interval range of only 0.35. This indicates the presence of weak double Debye dipole relaxation processes in CB. This may be due to the presence of trace metal elements in CB sample [53].

### 3.9. Microwave Absorbing Properties Analysis

According to the relevant theories [54,55], the RL is commonly used to characterize the absorption performance of absorbers (dB):

(2) $$RL=20log\left|\frac{Z_{in}-1}{Z_{in}+1}\right|,$$

(3) $$Z_{in}=\sqrt{\frac{\mu_{r}}{\varepsilon_{r}}}tanh\left(j\frac{2\pi fd}{c}\sqrt{\mu_{r}\varepsilon_{r}}\right)$$

where Z_in_ is the normalized impedance of the absorber, dimensionless; f is the electromagnetic wave frequency, Hz; c is the vacuum speed of light, m/s; j is an imaginary unit; d is the thickness of the absorber, m; μ_r_ = μ′− jμ″; and ε_r_ = ε′ − ε″.

**Figure 11:**
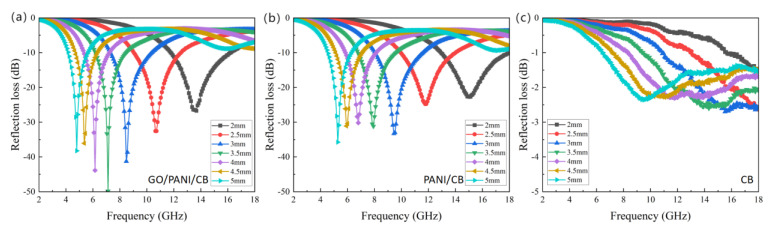
The calculated RL (dB) of GO/PANI/CB (**a**), PANI/CB (**b**) and CB (**c**) in the frequency range of 2–18 GHz.

The RL calculation results of GO/PANI/CB, PANI/CB, and CB are shown in Figure 11. The RL_min_ of GO/PANI/CB at 7.12 GHz is −49.83 dB with a thickness of 3.5 mm. The effective absorption bandwidth (<−10 dB) in the frequency range of 11.68–16.40 GHz is as high as 4.72 GHz with a thickness of 2 mm. The RL_min_ of PANI/CB at 5.28 GHz is −35.76 dB with a thickness of 5 mm, and it reaches 4.88 GHz with a thickness of 2 mm in the frequency range of 13.04–17.92 GHz. CB exhibits an RL_min_ of −2.7 dB at 15.52 GHz with a thickness of 3 mm.

The calculation results showed that CB itself had certain absorbing properties. The PANI/CB improved the absorption performance of CB by polymerizing PANI on the surface. The RL_min_ of PANI/CB was −35.76 dB, and its effective absorbing bandwidth was 4.88 GHz. With the addition of GO, GO/PANI/CB maintained its high effective absorbing bandwidth, and its reflection loss was as low as −49.83 dB, with a lower thickness. At the same time, the minimum reflection loss of GO/PANI/CB was less than that of PANI/CB in the thickness range of 2–5 mm, which reflected the superior absorption performance of GO/PANI/CB. This is consistent with the test results in the conductivity and electromagnetic properties sections. The excellent conductivity of GO/PANI/CB enhances its conductive loss capability. The double Debye relaxation phenomenon exhibits a better polarization relaxation loss capability. The combination of the two enhances the dielectric loss capability of the composites, which makes them exhibit excellent electromagnetic wave absorption properties.

**Figure 12:**
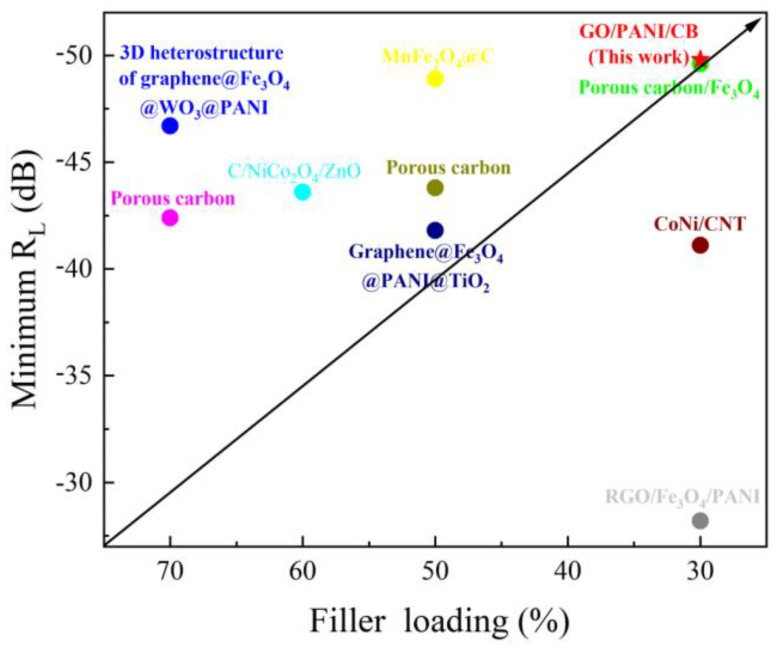
Reflection loss versus filler loading for the typical graphene/polyaniline based composites and carbon based materials reported in the recent literature.

Table 2 and Figure 12 presents a comparative analysis of the electromagnetic absorption index between GO/PANI/CB composite materials and other reported graphene/polyaniline based composite materials and carbon based materials. Obviously, compared with other graphene/polyaniline based composite materials and carbon materials, GO/PANI/CB composite materials have excellent properties, including excellent absorption strength and low filler loading.

**Figure 13:**
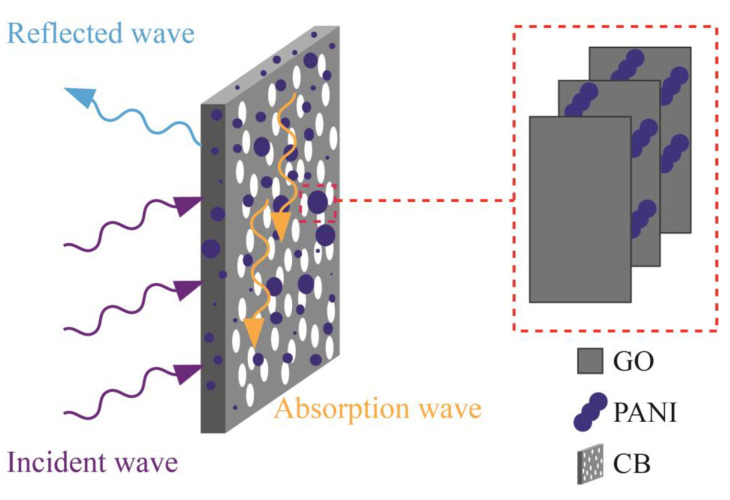
Schematic illustration of the microwave absorption mechanism for the GO/PANI/CB composite.

Based on the above analysis, the possible mechanism of GO/PANI/CB composite materials with microwave absorption performance can be summarized as shown in Figure 13. GO and PANI are arranged on the surface of CB in a sandwich like polymerization manner. The introduction of GO/PANI will result in dielectric loss [64,65]. It plays an important role in absorbing electromagnetic waves. The CB skeleton provides a pathway for the effective transmission and migration of electrons, leading to an increase in conductivity loss. The multiple reflections and scattering that occur in CB help to extend the propagation path of microwaves, thereby improving electromagnetic absorption performance.

## 4. Conclusions

This article described the preparation of a novel bamboo based composite material with microwave absorption properties through in situ polymerization. The oxygen-containing groups of GO react with PANI to form hydrogen bonds, leading to polymerization. It covers the surface of CB in the form of disordered clusters, forming an encapsulation of CB. By continuously optimizing the preparation process, the conductivity of GO/PANI/CB reached 2.17 ± 0.05 S/cm. The results shows that the RLmin of GO/PANI/CB at 7.12 GHz is −49.83 dB, with a thickness of 3.5 mm. The effective absorption bandwidth (<−10 dB) is as high as 4.72 GHz, with a frequency range of 11.68–16.40 GHz and a thickness of 2 mm.

The GO/PANI/CB has excellent conductivity loss and polarization relaxation loss capabilities. The combination of the two enhances the dielectric loss capability of the composite material. Meanwhile, the CB skeleton provides a pathway for effective electron transfer and migration, increasing the multiple reflections and scattering of electromagnetic waves. This makes GO/PANI/CB have excellent electromagnetic wave absorption performance. GO/PANI/CB has properties such as simple preparation, reproducibility and strong wave absorption, which meets the selection criteria of “thin, wide, light, and strong” absorbing materials. GO/PANI/CB has great potential to become a new type of microwave absorbing composite material.

## Figures and Tables

**Table 1:** The conductivity and composition of all samples.

Sample	GO (mg)	L-CSA (mol/L)	APS (mol/L)	CB (g)	Conductivity (S/cm)
PANI/CB	0	1	1	3	0.97 (±0.05)
GO-30	30	1	1	3	0.89 (±0.04)
GO-60	60	1	1	3	1.09 (±0.09)
GO/PANI/CB	90	1	1	3	2.17 (±0.05)
GO-120	120	1	1	3	1.61 (±0.07)
GO-150	150	1	1	3	1.18 (±0.04)
L-CSA-0.25	90	0.25	1	3	0.37 (±0.03)
L-CSA-0.5	90	0.5	1	3	0.72 (±0.03)
L-CSA-0.75	90	0.75	1	3	1.81 (±0.07)
L-CSA-1.25	90	1.25	1	3	1.76 (±0.03)
L-CSA-1.5	90	1.5	1	3	1.68 (±0.03)
APS-0.25	90	1	0.25	3	0.49 (±0.03)
APS-0.5	90	1	0.5	3	1.03 (±0.03)
APS-0.75	90	1	0.75	3	1.8 (±0.05)
APS-1.25	90	1	1.25	3	1.96 (±0.04)
APS-1.5	90	1	1.5	3	1.7(±0.04)
CB-1	90	1	1	1	1.7 (±0.03)
CB-2	90	1	1	2	1.91 (±0.04)
CB-4	90	1	1	4	1.5 (±0.06)
CB-5	90	1	1	5	0.92 (±0.05)
CB-6	90	1	1	6	0.43 (±0.03)

**Table 2:** Electromagnetic absorption indices of graphene/polyaniline based composites and carbon-based materials in recent years.

Sample	RL_min_ (dB)	Minimum Peak Position (GHz)	Filler Loading (%)	Ref.
RGO/Fe_3_O_4_/PANI	–28.2	5.4	30	[26]
Graphene@Fe_3_O_4_@PANI@TiO_2_	–41.8	14.4	50	[56]
3D heterostructure of graphene@Fe_3_O_4_@WO_3_@PANI	–46.7	9.4	70	[57]
CoNi/CNT	−41.1	8.9	30	[58]
Porous carbon/Fe_3_O_4_	−49.6	15.9	30	[59]
Porous carbon	−42.4	8.9	70	[60]
MnFe_2_O_4_@C	−48.9	0.8	50	[61]
Porous carbon	−43.8	8.3	50	[62]
C/NiCo_2_O_4_/ZnO	−43.6	11.6	60	[63]
GO/PANI/CB (This work)	−49.8	7.12	30	

## Data Availability

Data will be available on request.
